# Impact of Platelet Glycoprotein Ia/IIa C807T Gene Polymorphisms on Coronary Artery Aneurysms of KD Patients

**DOI:** 10.1155/2021/4895793

**Published:** 2021-02-23

**Authors:** Wei Li, Lei Pi, Jia Yuan, Xueping Gu, Zhouping Wang, Yunfeng Liu, Qiulian Deng, Yanfei Wang, Ping Huang, Li Zhang, Xiaoqiong Gu

**Affiliations:** ^1^Department of Cardiology, Guangzhou Women and Children's Medical Center, Guangzhou Medical University, Guangzhou 510120, China; ^2^Department of Clinical Biological Resource Bank, Guangzhou Institute of Pediatrics, Guangzhou Women and Children's Medical Center, Guangzhou Medical University, Guangzhou 510623, China; ^3^Department of Blood Transfusion and Clinical Lab, Guangzhou Institute of Pediatrics, Guangzhou Women and Children's Medical Center, Guangzhou Medical University, Guangzhou 510623, China; ^4^Department of Clinical Lab, Guangzhou Institute of Pediatrics, Guangzhou Women and Children's Medical Center, Guangzhou Medical University, Guangzhou 510623, China

## Abstract

**Background:**

Kawasaki disease (KD) is a systemic vasculitis of unknown etiology in children. Coronary artery abnormalities are the most common complications of KD. Recent evidence showed that genetic polymorphisms may lead to susceptibility to KD. Genetic variants in platelet glycoprotein have been reported to be associated with coronary artery disease. The aim of the present study is to investigate the correlation between the role of platelet glycoprotein and coronary artery aneurysms in KD patients.

**Methods:**

We did a case-control study that enrolled 818 KD patients and 1401 healthy children with the same age and sex from January 2013 to December 2016. Analysis of single-nucleotide polymorphism (rs1126643) of the platelet glycoprotein Ia/IIa C807T was performed by multiplex polymerase chain reactions in this study.

**Results:**

A significant difference in the genotype distribution between KD cases and controls was observed for the glycoprotein Ia/IIa C807T (rs1126643) polymorphism (*p*=0.026). Compared with the healthy children, the rs1126643T allele carriers had odds ratio (OR) of 0.63 for developing KD (TT vs. CC: adjusted OR = 0.62, 95% confidence interval (CI) = 0.43–0.88,*p*=0.0078; TT vs. CT/CC: adjusted OR = 0.63, 95% CI = 0.44–0.889,*p*=0.0093). Furthermore, we also found that children less than 60 months of age and female patients with rs1126643 T allele carriers had an adjusted OR of 0.66 (95% CI = 0.46–0.95) for noncoronary artery aneurysm patients (*p*=0.0242). Single-nucleotide polymorphism rs1126643 TT seems to represent a protective factor against KD in coronary artery aneurysm formation in multivariate analysis.

**Conclusions:**

The platelet glycoprotein Ia/IIa T allele carriers may have a protective effect on the risk of coronary artery aneurysms of KD patients, especially in females and children aged less than 60 months. These results may provide evidence for platelet glycoprotein Ia/IIa gene polymorphisms in the pathogenesis of KD patients.

## 1. Introduction

Kawasaki disease (KD) is an acute vasculitis that affects young children less than 60 months of age and KD has been diagnosed in many countries; however, it is more common in Asia [1, 2]. Its etiology and pathogenesis remain unknown [3, 4]. The most serious complications of KD are coronary artery aneurysms (CAAs). Previous studies indicated the interindividual variability in susceptibility to KD. The different incidence rates of KD among ethnicities are related to genetic factors [5–7]. Previously, our research group had found the correlation between the polymorphisms of the genes thromboxane A2 receptor, interleukin-1-beta, and KD with coronary artery abnormalities [8, 9]. In summary, these previous studies suggested that genetic susceptibility may contribute to KD.

Platelet glycoprotein (GP) Ia/IIa of the coding region of the platelet receptor for collagen is known as a key member of the integrin family. Platelet GP Ia/IIa can mediate interactions among cells or between cells and the extracellular matrix [10–12]. These proteins can aid white blood cells and platelets in adhering to the vascular endothelium. So they play important roles in physiological and pathological processes of inflammatory reaction, immune response, atherosclerosis, and thrombosis [13–16]. The platelet GP Ia/IIa is a major collagen receptor on the platelet [17]. Moreover, platelet activation and aggregation play a crucial role in the progression of KD [18]. It remains unclear whether the role of GP Ia/IIa C807T (rs1126643) gene polymorphisms is related to KD with CAAs susceptibility.

There have been a few studies on the association between GP Ia/IIa C807T (rs1126643) gene polymorphisms and acute coronary artery syndromes [17, 19–21]. However, the role of GP Ia/IIa C807T (rs1126643) and their genetic variants in KD remains unknown, and changes in platelet function related to the presence of this polymorphism in KD have not been shown. Accordingly, the aim of the present study is to evaluate the association of gene polymorphisms in GP Ia/IIa C807T (rs1126643) with susceptibility of KD in a larger sample of patients admitted to Guangzhou Women and Children's Medical Center.

## 2. Methods

### 2.1. Study Protocol

After the approval of the Institutional Review Board and the Ethics Committee of the Medical Center, all informed consents were obtained from all patients or participant's guardian. The following baseline characteristics were collected: age, gender, and coronary artery involvement. We aimed to investigate the association between GP Ia/IIa C807T (rs1126643) polymorphisms and the risk of CAAs in KD. We randomly selected 818 consecutive KD patients admitted to Guangzhou Women and Children's Medical Center from January 1, 2013, to December 31, 2016. All patients were treated with intravenous immunoglobulin and aspirin. The inclusion criteria for study subjects were as follows: (i) patients were ≤18 years old and only complete and incomplete KD cases in accordance with the 2004 American Heart Association guidelines of KD were enrolled, and (ii) initial onset of the disease. Exclusion criteria were as follows: (i) serious infection, (ii) allergy disease, and (iii) collagen disease. Additionally, 1401 healthy controls with the same age and sex were selected from children who had a physical examination without a history of thromboembolic events or a tendency to bleed. We used echocardiography for defining coronary artery abnormalities in the evaluation of patients with KD. Definition of CAAs [2] was defined as small (<5 mm internal diameter), medium (5–8 mm internal diameter), or giant (>8 mm internal diameter).

### 2.2. Genetic Analysis of Single-Nucleotide Polymorphisms

The single-nucleotide polymorphisms (SNPs) in this study were chosen from the National Center for Biotechnology Information dbSNP database based on the three criteria [8]: (i) a minor allele frequency of ≥5% in Southern Chinese offspring reported in Hap Map; (ii) located in the gene regulatory region; and (iii) influencing the transcription factor binding site activity or microRNA binding site activity in the putative promoter region or changing amino acids in the exons.

A peripheral blood sample was collected from each individual to perform genetic analysis. According to the manufacturer's instructions, the genomic DNA Extraction Kit (Tiangen, China) was used to extract leukocyte genomic DNA, which was preserved at −80°C until later use. The concentration and quality of genomic DNA were measured using a nucleic acid quantifier. According to the literature review and the criteria of SNPs [8], genomic DNA samples were amplified by polymerase chain reactions (PCRs) with the following gene-specific primer pairs for rs1126643: forward 5′-TGATTGTAGCAACATCCCAGACA-3′/reverse 5′-ATGAAAACATTGGCCTATTAGCACC-3′. Primers are reported in Table 1. The PCR was carried out in accordance with the standard operating procedure using a Gene Amp PCR System 9700 (Thermo Fisher Scientific). The PCR products were subjected to massive parallel sequencing on an Ion Proton system (Life Technologies).

### 2.3. Statistical Analysis

Statistical analyses were performed with Statistical Analysis System software (version 9.3; SAS Institute, Cary, NC). Data were expressed as mean ± standard deviation. The chi-squared test was used to assess differences between the KD cases and the healthy controls. Genotype and allele frequencies were calculated with the direct gene counting method. Tests of deviations from Hardy–Weinberg equilibrium (HWE) were applied for the samples using the goodness-of-fit chi-squared test. We calculated crude odds ratios (ORs) and 95% confidence intervals (CIs) by standard methodology as estimates of the relative risk for KD for the GP Ia/IIa C807T (rs1126643). Adjustment for age and sex was done and unconditional logistic regression was used to analyze the association between rs1126643 and KD susceptibility. All *p* values in the current study were two-sided, and a *p* value of less than 0.05 was considered as statistical significance.

## 3. Results

### 3.1. Basic Characteristics

Table 2 summarizes the demographic characteristics of the KD patients and healthy controls. The mean age was 29.98 ± 25.27 months for KD patients and 26.78 ± 26.15 months for the controls. The proportions of males were 67.48% and 66.45%, respectively. No statistical differences in age and gender were observed between KD patients and the controls. Of the KD cases, according to the 2013 Japanese Circulation Society guidelines with coronary artery lesions criteria [2], in our study, 14.18% of KD patients developed CAAs and 85.82% of patients had non-CAAs.

### 3.2. Correlations between Platelet Glycoprotein Ia/IIa C807T (rs1126643) Polymorphisms and KD Susceptibility

The frequency distribution of rs1126643 polymorphisms in the KD patients and healthy controls is presented in Table 3. The distribution of rs1126643 genotype within both the control children and KD patient groups reached Hardy–Weinberg equilibrium (*p*=0.9626). A significant difference in the distribution of the rs1126643 genotype and allele frequencies was observed between the two groups (*p*=0.026). After adjustments for age and sex, Comparing with CC genotype, T allele carriers had ORs of 0.63 for developing KD (TT vs. CC: adjusted OR = 0.62, 95% CI = 0.43–0.88,*p*=0.0078; TT vs. CT/CC: adjusted OR = 0.63, 95% CI = 0.44–0.89, *p*=0.0093). Specifically, rs1126643 T allele carriers had a higher probability of a protective effect against KD.

### 3.3. Stratified Analysis

As shown in Table 4, we further explored rs1126643 gene polymorphism association with the combined effects of protective genotypes in KD patient susceptibility in stratification analysis by age, gender, and coronary artery outcomes. Compared to the CC/CT genotype, rs1126643 T allele carriers were more predominant for children less than 60 months of age (adjusted OR = 0.64, 95% CI = 0.44–0.93) and females (adjusted OR = 0.40, 95% CI = 0.20–0.79). In terms of coronary artery outcomes, we observed that rs1126643 TT genotype had a significantly decreased risk of KD patients with non-CAAs (adjusted OR = 0.66, 95% CI = 0.46–0.95), suggesting the protective effect of this SNP against KD in CAA formation. No significant difference was observed in allele frequencies in KD patients with CAAs.

## 4. Discussion

In our study, we revealed the association between the GP Ia/IIa C807T (rs1126643) and KD susceptibility in Chinese children, in which the rs1126643 T allele carriers may have a protective effect in KD children. Furthermore, in the stratified analysis, if the age of onset is less than 60 months and female children, we observed that carriers of the rs1126643T allele carriers had a more protective of non-CAAs in KD than those CC/CT genotypes.

The platelet GP Ia/IIa C807T gene locates on chromosome 5q23-31. The C807T polymorphism (rs1126643) was previously associated with some individual variation in platelet expression levels of GP Ia/IIa, the preferential platelet receptor for collagen, which plays a crucial role in platelet adhesion and activation [12]. It is reported that GP Ia/IIa C807T gene polymorphisms were a risk factor for patients in myocardial infarction and stroke [16, 21, 22]. For these reasons, the study of allelic variants of platelet glycoprotein genes may be useful to interpret the pathophysiology of coronary artery disease [23–25].

Although KD was first reported over 50 years, the etiology of KD remains unknown. However, the high rate of KD in children of Asian ethnicity showed that KD was associated with genetic susceptibility among different populations [7, 26]. There may be a higher risk of coronary artery abnormalities if the KD diagnosis is delayed. Previous studies [21, 27] had found the GP Ia/IIa C807T (rs1126643) polymorphism affects GP Ia/IIa expression. The genes were associated with the risk of cardiovascular disease. Santoso et al. [21] reported that the GP Ia/IIa C807T allele is associated with the incidence of myocardial infarction in younger patients. On the other hand, Tsantes et al. [27] found that GP Ia/IIa C807T polymorphism of the GP Ia gene is not a significant risk factor for coronary artery disease. In a word, previous studies [22, 26–28] demonstrated the correlation between coronary artery disease and some single-nucleotide polymorphisms, especially the genes encoding for platelet glycoprotein.

There is growing evidence that genetic polymorphisms are important determinants of CAAs in KD, which could result in predispositions to adverse outcomes [29–31]. However, there is less evidence about the relation between platelet receptor gene polymorphisms and KD, especially coronary artery abnormality. So we systematically investigated the association between GP Ia/IIa C807T (rs1126643) gene polymorphisms and the effect of KD in a Chinese population. Our study showed that a significant difference in genotype distribution between KD patients with rs1126643 T allele carriers was observed compared with the healthy children, indicating the impact of T allele carriers in KD patients. Moreover, we also found that children with rs1126643 T allele carriers had an adjusting OR of 0.66 for noncoronary artery aneurysms of KD patients (*p*=0.0242), suggesting the protective effect of this genotype against KD in CAA formation. So we can hypothesize that KD is associated with the function of platelet glycoprotein Ia/IIa. Furthermore, at the stratified analysis of logistic regression models, our data also showed that GP Ia/IIa C807T (rs1126643) protective genotype was more evident in children ≤60 months of age and females. In a word, the protective role of rs1126643 T allele carriers may decrease the risk of KD in females and children younger than 60 months of age in this study.

KD is involved in systemic vasculitis that affects small- and medium-sized vessels and especially affects the coronary arteries leading to CAAs, which is the major complication of KD. Thrombocytosis is a characteristic feature of KD [1]. Timely diagnosis and treatment with high-dose IVIG and aspirin can decrease coronary artery lesion formation in patients with KD in the acute phase [32]. Moreover, GP Ia/IIa is a major collagen receptor on the platelet, which may serve as an important factor of platelet adhesion and activation in the development of KD. These results confirm the potential role of SNP of genes for GP Ia/IIa in the determinism of KD as an independent protective factor.

There were several limitations of this study. First, we included only the GP Ia/IIa C807T (rs1126643) allele. Other GP Ia/IIa gene polymorphisms were not involved in this study. Second, the sample size in the current study was not sufficiently large. Third, this is a single-center study. And all subjects recruited had the same ethnicity (Chinese). Because of the limitations of this study, more GP Ia/IIa alleles and large sample are needed to confirm the roles of gene in KD with CAAs susceptibility.

## 5. Conclusions

The platelet GP Ia/IIa C807T (rs1126643) T allele carriers may have a protective effect on the risk of CAA formation of KD patients less than 60 months of age in a Chinese population. These results may provide evidence for GP Ia/IIa C807T (rs1126643) gene polymorphisms in the pathogenesis of KD patients. Our findings need to be confirmed in a larger, prospective study that includes KD patients from different populations.

## Figures and Tables

**Table 1:** Single-nucleotide polymorphism of platelet glycoprotein Ia/IIa analysed and relative primers used in the study.

SNP ID	Nucleotide change	Amino acid change	Primers	*T*_*a*_ (°C)
rs1126643	C-T	Phe253leu	5′-TGATTGTAGCAACATCCCAGACA-3′	60

SNP ID: reference single-nucleotide polymorphism ID (https://www.ncbi.nlm.nih.gov/snp/); *T*_*a*_: annealing temperature.

**Table 2:** Baseline and clinical characteristics in Kawasaki disease cases and healthy controls.

Variable	KD cases	Healthy controls	*p* value^a^
Age range (months)	1–166	1–144	
Mean ± SD	29.98 ± 25.27	26.78 ± 26.15	
Age ≤ 60 months	729 (89.12%)	1276 (91.08%)	0.1345
Age > 60 months	89 (10.88%)	125 (8.92%)	

*Gender*			
Male	552 (67.48%)	931 (66.45%)	0.6191
Female	266 (32.52%)	470 (33.55%)	

*Severity of coronary artery lesions*			
NCAAs	702 (85.82%)		
CAAs	116 (14.18%)		

^a^Two-sided *χ*^2^ test for distributions between Kawasaki disease patients and healthy controls. NCAAs: noncoronary artery aneurysms; CAAs: coronary artery aneurysms; KD: Kawasaki disease.

**Table 3:** Genotype frequency distribution of platelet glycoprotein Ia/IIa C807T (rs1126643) polymorphism in Kawasaki disease cases and healthy controls.

Genotype	KD cases (*n* = 818)	Controls (*n* = 1401)	*p* value^a^	OR (95% CI)	*p* value	Adjusted OR (95% CI)	Adjusted *p* value^b^
rs1126643	HWE = 0.9626						
CC	429 (52.44%)	695 (49.61%)	—	1.00	—	1.00	—
CT	342 (41.81%)	583 (41.61%)	—	0.95 (0.79–1.14)	0.5787	0.95 (0.79–1.14)	0.5784
TT	47 (5.75%)	123 (8.78%)	—	0.62 (0.43–0.89)	**0.0085**	0.62 (0.43–0.88)	**0.0078**
Additive	—	—	**0.0260**	0.86 (0.75–0.99)	**0.0347**	0.86 (0.75–0.99)	**0.0331**
Dominant	389 (47.56%)	706 (50.39%)	0.1970	0.89 (0.75–1.06)	0.1972	0.89 (0.75–1.06)	0.1941
Recessive	771 (94.25%)	1278 (91.22%)	**0.0082**	0.63 (0.45–0.90)	**0.0101**	0.63 (0.44–0.89)	**0.0093**
C	1200 (73.35%)	1973 (70.41%)	**0.0361**	1.00	**—**	1.00	**—**
T	436 (26.65%)	829 (29.59%)	**—**	0.87 (0.76–0.99)	**0.0367**	0.86 (0.75–0.99)	**0.0351**

^a^Two-sided *χ*^2^ test for distributions between Kawasaki disease patients and healthy controls. ^b^Adjusted for age and sex status in logistic regression models. OR: odds ratio; CI: confidence interval; KD: Kawasaki disease.

**Table 4:** Stratification analysis of platelet glycoprotein Ia/IIa C807T(rs1126643) gene polymorphism in Kawasaki disease cases and healthy controls.

Variable	rs1126643(cases/controls)		*p* value^a^	OR (95% CI)	*p* value	Adjusted OR (95% CI)	Adjusted *p* value^b^
	CC/CT	TT					
*Age (months)*							
≤60	688/1167	41/109	**0.0148**	0.64 (0.44–0.93)	**0.0177**	0.64 (0.44–0.93)	**0.0176**
>60	83/111	6/14	0.2613	0.57 (0.21–1.56)	0.2744	0.57 (0.21–1.55)	0.2719

*Gender*							
Male	516/853	36/78	0.1898	0.76 (0.51–1.15)	0.1957	0.76 (0.51–1.15)	0.1907
Female	255/425	11/45	**0.0052**	0.41 (0.21–0.80)	**0.0094**	0.40 (0.20–0.79)	**0.0085**

*Coronary artery outcomes*							
NCAAs	660/1278	42/123	**0.0216**	0.66 (0.46–0.95)	**0.0254**	0.66 (0.46–0.95)	**0.0242**
CAAs	111/1278	5/123	0.0701	0.47 (0.19–1.17)	0.1039	0.46 (0.18–1.15)	0.0973

^a^ Two-sided *χ*^2^ test for distributions between Kawasaki disease patients and healthy controls. ^b^Adjusted for age and sex status in logistic regression models. NCAAs: noncoronary artery aneurysms; CAAs: coronary artery aneurysms; OR: odds ratio; CI: confidence interval; KD: Kawasaki disease.

## Data Availability

The data used to support the findings of this study are available from the corresponding author upon request.
